# A Novel Approach Using LuxSit-i Enhanced Toehold Switches for the Rapid Detection of *Vibrio parahaemolyticus*

**DOI:** 10.3390/bios14120637

**Published:** 2024-12-21

**Authors:** Xiaodan Kang, Chen Zhao, Shuting Chen, Shuran Yang, Xi Zhang, Bin Xue, Chenyu Li, Shang Wang, Xiaobo Yang, Chao Li, Zhigang Qiu, Jingfeng Wang, Zhiqiang Shen

**Affiliations:** 1 Military Medical Sciences Academy, Tianjin 300050, China; 13849918731@163.com (X.K.); zhaochen212@126.com (C.Z.); 18701824329@163.com (S.C.); 13502118928@163.com (S.Y.); zhangxi0820@126.com (X.Z.); xue_bin04@163.com (B.X.); nk_lcy710430@hotmail.com (C.L.); wsh847@163.com (S.W.); 18072712080@163.com (X.Y.); lc6628@163.com (C.L.); zhigangqiu99@gmail.com (Z.Q.); 2 College of Food Science and Technology, Shanghai Ocean University, Shanghai 201306, China

**Keywords:** *Vibrio parahaemolyticus*, toehold switch, LuxSit-i, on-site detection

## Abstract

*Vibrio parahaemolyticus* (*V. parahaemolyticus*) is a significant concern, as it can cause severe infections and hemolytic trauma. Given its prevalence in seawater and coastal seafood, it poses a substantial risk as a foodborne pathogen. Biosensor-based detection technology has been continuously evolving, and toehold switches have emerged as a promising area within it, especially in the detection of RNA viruses. Here, we have developed a cell-free toehold switch sensor for *V. parahaemolyticus* detection. Traditional toehold switch detection methods usually use green fluorescent protein (GFP) or enzyme LacZ as the output signal, with an incubation time as long as 2 h, and are also mainly applied to the detection of RNA viruses. In this study, we introduced a novel, artificially designed luciferase (LuxSit-i) as an output signal and constructed toehold switches with two different output signals (sfGFP, LuxSit-i), aimed at reducing the incubation time of toehold switches. Moreover, to further improve the detection process, we separately utilize recombinase polymerase amplification (RPA) and nucleic acid sequence-based amplification (NASBA) to amplify dead and live bacterial suspensions for detection and attempt to distinguish between dead and live bacteria. This study provided a convenient, rapid, and accurate method for the on-site detection of *V. parahaemolyticus*, especially beneficial for resource-limited settings. By eliminating the requirement for specialized facilities and personnel, this system has the potential to be a valuable tool in improving public health responses, especially in developing regions.

## 1. Introduction

In recent years, the emergence of toehold switches has laid a novel and general foundation for future detection methods. Alexander A. Green has designed a type of riboregulators that are created anew. These riboregulators can activate protein translation at the post-transcriptional level through mechanisms utilized in artificial systems instead of natural ones [1]. The presence of a target gene in the system is determined by adding toehold switch sensors to the cell-free system to the output signal. It is mainly used for RNA virus detection, and so far, for many viruses like Zika [2], Coronaviruses [3], and SARS-CoV-2 [4], detection methods have been developed based on toehold switches; the results are outstanding. In general, the combination of a toehold switch and nucleic acid sequence-based amplification (NASBA) lowers the detection limit to aM [5,6]. However, when it comes to bacteria whose fundamental genetic substance is DNA, there are no reports on toehold switch-based detection up to now. In addition, in the existing detection systems, the output signal often involves relatively large protein molecules. This characteristic of the output signal has led to a prolonged detection time, and currently, there is no effective solution to this problem. This situation has restricted the development and application of toehold switch-based detection methods in bacteria-related detection, highlighting the need for further research and innovation in this area.

Non-cellular biosensors mainly output signals in optical or electrochemical forms. Reporter genes are typically classified into fluorescence (e.g., GFP), colorimetry (e.g., LacZ), and luminescence (e.g., Lux, Luc) types [7,8]. In toehold switch systems, GFP fluorescence and LacZ-induced color changes are common signal outputs, with a relatively long incubation time of about 2 h [4,9]. Notably, luciferase has not been reported as a signal output in a toehold switch, mainly due to its cofactor dependence and non-specificity. However, a newly designed artificial luciferase, LuxSit-i, has characteristics such as high sensitivity, high specificity, small size, and low background signals, showing potential for use in toehold switches. Compared with GFP and LacZ, LuxSit-i is smaller and requires less in vitro synthesis time, which may potentially reduce the incubation time of the toehold switch. This paper aims to explore the application of LuxSit-i in toehold switch systems.

*Vibrio parahaemolyticus* (*V. parahaemolyticus*) is a Gram-negative, non-spore-forming, flagellated, motile, facultative anaerobic halophilic bacterium [10], and foodborne diseases caused by it are mainly related to aquatic and marine products. Its main virulence factors are encoded by specific genes (thermolabile hemolysin: tlh) [11,12]. Currently, various techniques such as PCR, the loop-mediated iso-thermal amplification technique (LAMP), gene hybridization, and immunological methods have been used for its detection [13]. Each method has its own advantages and disadvantages. For example, immunological methods are highly specific and sensitive but have high costs and long-term antibody preparation in the preliminary stage [14], while molecular biology-based methods mostly require laboratories, skilled operators, and expensive equipment [13].

Among the various detection methods, amplification technologies play a vital role. When it comes to targeting DNA, recombinase polymerase amplification (RPA) has shown its potential. In this study, RPA was selected to amplify the conserved sequences of *V. parahaemolyticus*. It is known that RNA is only present in living cells, in contrast to DNA, which can be present in both living and dead cells [15,16]. Based on this characteristic, nucleic acid sequence-based amplification (NASBA), which is more relevant for the detection of living cells, as it targets RNA, has been chosen as a preamplification method. Signal output is another important aspect in detection systems. In this study, superfold green fluorescent protein (sfGFP) and LuxSit-i were utilized as signal outputs. SfGFP is a well-known signal generating molecule, and LuxSit-i, a newly designed artificial luciferase, holds great promise due to its high sensitivity, high specificity, small size, and low background signals.

Toehold switches have recently emerged as an innovative approach in detection methods. By combining the toehold switch with NASBA and RPA separately, we aimed to not only achieve discrimination detection between live and dead *V. parahaemolyticus* bacteria but also to shorten the overall detection time. This could potentially overcome some of the limitations of existing detection methods for this pathogen and contribute to more effective food safety monitoring and disease prevention.

## 2. Materials and Methods

### 2.1. Toehold Switch Sensor In Silico Design

Toehold switch sensor was created using the B version as outlined in Pardee’s previous description [2]. In essence, the toehold switch consisted of a toehold sequence, a stem-loop structure, and a 21-nucleotide linker that connected to the reporter gene. The toehold sequence was designed to be a 25-nucleotide segment that complemented the target sequence. The *V. parahaemolyticus* tlh gene sequence was employed to generate suitable sequences with optimal secondary structures following the toehold switch sensor design protocol [17] specified in the design module of the NUPACK nucleic acid sequence design tool [18]. The remaining components of the sensor were adapted from the conserved regions cited in Pardee’s work [2]. SfGFP [4] and LuxSit-i [19] were used as reporter genes, respectively. All candidate sequences were shown in Table S1. Reporter gene sequence was shown in Table S3.

### 2.2. Toehold Switch Sensor Construction

The toehold switch was amplified from a template plasmid via PCR. The template plasmid was constructed based on pCOLADuet-1 and contained a kanamycin resistance gene. The toehold switch sequences were inserted between the T7 promoter and terminator using homologous recombination. All DNA segments for insertion were synthesized by GENEWIZ (Suzhou, China). The recombinant plasmids were introduced into *E. coli* TOP10 cells and cultured in LB medium containing kanamycin (60 μg/mL) at 37 °C. After transformation, monoclonal colonies were selected to verify the correctness of the sequence through Sanger sequencing. The liner switch was amplified by PCR using Phusion High-Fidelity DNA polymerase (M0530L, NEB, New England, Ipswich, MA, USA) and purified via QIAquick PCR Purification Kit (28106, QIAGEN, Dusseldorf, Germany). All primer sequences were shown in Table S2. The construction of trigger plasmid is consistent with our previous work [20].

### 2.3. Toehold Switch Sensor Select in Whole Cell

The switch template plasmid and trigger plasmid were chemically co-transformed into *E. coli* BL21, respectively. Simultaneously, control groups were generated by the switch template plasmid transformation only. A single colony was selected from each plate and incubated in luria bertani (LB) medium at 37 °C with suitable antibiotics using a 150 rpms orbital shaker overnight. Subsequently, the cells were resuspended in PBS. The overall signal emission was quantified using plate reader (M5 SpectraMax, Molecular Devices, Framingham, MA, USA). The ON/OFF value was determined by calculating the ratio of the sensor’s value to corresponding control groups’ value. All the reported values subtracted the blank value derived from the reaction lacking both the toehold switch sensors and trigger.

### 2.4. Cell-Free Reaction Assembly and Incubation

Cell-free reaction was validated according to PURExpress In Vitro Protein Synthesis Kit (E6800S, NEB, New England, Ipswich, MA, USA) protocol. Briefly, cell-free reaction mixture consisted of 8 µL solution A (E6800S, NEB, New England, Ipswich, MA, USA), 6 µL solution B (E6800S, NEB, New England, Ipswich, MA, USA), 0.4 µL Rnase inhibitor (M0314L, NEB, New England, Ipswich, MA, USA), toehold switch liner DNA (40 nM or 10 nM), RNase-free water up to 20 μL. Total fluorescence was measured by M5 SpectraMax (Molecular Devices, Framingham, MA, USA) for 2 h at 37 °C to monitor sfGFP expression at 538 nm. For LuxSit-i as the signal output, the incubation lasted for 0.5 h at 37 °C, and then, Luciferase substrate diphenylterazine (DTZ) (344940-63-2, MCE, Monmouth Junction, NJ, USA) was added into the mixture. Total chemiluminescence was immediately measured by M5 SpectraMax (Molecular Devices, Ipswich, MA, USA).

### 2.5. RPA Reactions

The overall approach for the RPA reactions was validated according to TwistAmp basic (TABAS03KIT, TwistDx, Cambridge, UK) protocol. Briefly, RPA reaction mixture consisted of 2.4 µL forward primer, 2.4 µL reverse primer, 29.5 µL primer free rehydration buffer, 3.2 µL template DNA (trigger DNA), and ddH_2_O up to 47.5 µL. Vortex and spin briefly the reaction mix before added to a TwistAmp basic reaction for blending. Next, add 2.5 µL of 280 mM magnesium acetate and mix well to start reaction at 37 °C for 20 min. The final DNA concentration was determined by Nanodrop (Thermo Fisher Scientific, Waltham, MA, USA). All primer sequences were shown in Table S2.

### 2.6. NASBA Reactions

The general approach utilized for the NASBA reactions was derived from a technique outlined by our previous work [20]. Briefly, each reaction consisted of 50 mM Tris-HCl, 8 mM MgCl_2_, 10 mM Dithiothreitol, 75 mM KCl (pH = 8.5), 7.5% dimethyl sulfoxide (DMSO), 0.25 mM each dNTP, 0.5 mM each NTP, 0.25 μM of each NASBA primer, and template RNAs and RNase-free water up to 20 μL. The template RNAs’ secondary structure was initially disrupted at a temperature of 65 °C for a duration of 5 min, which was subsequently followed by incubation at 41 °C for another 5 min. Then, the enzyme mixture (including 0.25 µg · µL^−1^ BSA, 40 U T7 RNA Polymerase, 10 U AMV Reverse Transcriptase, 0.5 U RNase H) was added to make a final volume of 20 μL, and thereafter, the mixture was incubated at 41 °C for 2 h. The final RNA concentration was determined by Qubit 4 fluorometer (Thermo Fisher Scientific, Waltham, MA, USA). All primer sequences were shown in Table S2.

### 2.7. V. parahaemolyticus Detection

*V. parahaemolyticus* (ATCC, 17802) was maintained in the laboratory. *V. parahaemolyticus* was cultured with brain–heart infusion (BHI) supplemented with sodium chloride. To obtain dead *V. parahaemolyticus* samples, *V. parahaemolyticus* was treated with 75% alcohol for 20 min. Before cell-free detection, live and dead bacteria were boiled at 95 °C for 5 min to obtain lysate. The lysate was then added to the RPA and NASBA reactions for next detection. The bacterial concentration (colony-forming units, CFU) was determined by plating serial dilutions of the bacterial suspension onto agar plates. After overnight incubation at 37 °C, colonies were counted, and the CFU concentration was calculated using the following formula:

$$CFU/mL=\frac{Number\;of\;colonies}{Dilution\;factor*Volume\;plated\;(mL)}$$

### 2.8. Specificity and Sensitivity Assay

To assess the sensitivity, varying concentrations of trigger were introduced to the cell-free reactions for fluorescence or chemiluminescence measurement. Regarding the sensitivity after isothermal amplification, different concentrations of trigger were utilized as template amplification reactions, and subsequently, 1 μL of amplification products were utilized in the cell-free toehold switch sensor.

To evaluate specificity of the toehold switch sensor, other homologous species and common pathogenic bacteria, including *vibrio fluvialis*, *vibrio cholerae*, *vibrio vulnificus*, *escherichia coli*, *staphylococcus aureus*, *listeria monocytogenes*, *klebsiella pneumoniae*, *pseudomonas aeruginosa*, and *enterobacter sakazaki*, were selected as potential cross-reactive species in the cell-free toehold switch sensor. The pre-treatment was the same as *V. parahaemolyticus* detection.

## 3. Results

### 3.1. Principle of the Toehold Switch Sensor for V. parahaemolyticus Detection

Toehold switch sensors are programmable synthetic riboregulators that control the reporter translation via the binding of a trans-acting trigger RNA [21]. The switches contain a hairpin structure that blocks gene translation in cis by sequestration of the ribosome binding site (RBS) and start codon (AUG). Upon a switch binding to a complementary trigger RNA, blocked RBS and AUG are relieved, activating reporter translation [1]. The sensors can be designed to regulate the reporter expression, for instance, sfGFP, which produces green fluorescence, or LacZ, which catalyzes substrate oxidation, resulting in a color change. A toehold switch sensor for the detection of *V. parahaemolyticus* was designed based on the B version described by Pardee previously within this experiment [2]. The Tlh gene in the *V. parahaemolyticus*-corresponding RNA sequence was selected as trigger RNA here. Using NUPACK [21,22] to calculate the ensemble defect, design switch, and trigger, potential candidates with lower ensemble defects were chosen for the construction of toehold switch sensors.

**Figure 1:**
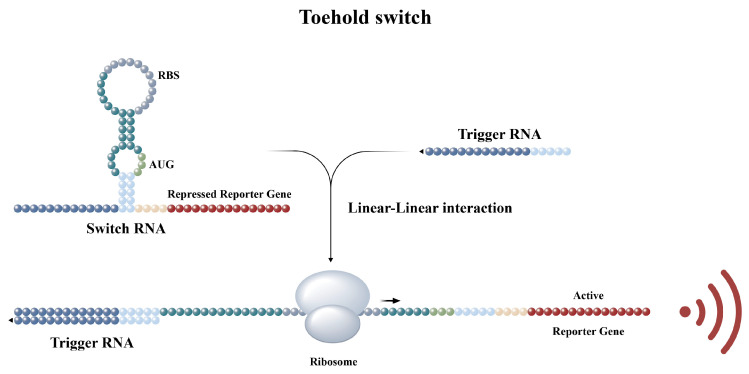
A schematic of the toehold switch sensor. RBS: ribosome binding sites. AUG: start codon.

The toehold switch sensor for *V. parahaemolyticus* detection is shown in Figure 1. In the absence of trigger RNA, the hairpin unit in the sensor hides the RBS and AUG, repressing translation [1]. When trigger RNA binds to the toehold region of the switch RNA, a new double stranded sequence forms [23], exposing the AUG [24] and RBS [6] and activating reporter expression [25]. Each sensor varies only in the trigger binding (dark blue) and lower stem (light blue) regions (Figure 1), while the other sequences remained unchanged, enabling a pathogen-detection-oriented design. As a result, eight different switch chains were constructed, each having different binding affinities with the trigger [6,21]. Two reporters were used for different signal outputs. SfGFP, shorter and more efficient in synthesis than LacZ, was the first reporter. It can produce a visible green fluorescence signal without substrates under excitation light [1]. LuxSit-i, an artificial luciferase created by Yeh’s team [19], was the second reporter. Native luciferases have limitations like scarcity in identification and poor recognition of synthetic luciferins [26,27], so their use as a signal output has been less developed compared to fluorescent proteins [28]. However, LuxSit-i is small, stable, substrate specific, and cofactor free and has a high catalytic efficiency (k_cat_/K_m_ = 10^6^ M^−1^ s^−1^) for DTZ [29] luminescence, with a visible signal and a photon flux (photons per second) 38% greater than native reniformis luciferase (RLuc) [19]. It has a shorter gene sequence and smaller protein molecular weight than sfGFP. This is the first attempt to apply luciferases in toehold switches, which broadens the range of signal output options and may open up new possibilities for more sensitive and specific pathogen detection in the future.

### 3.2. Sensor Sequence Design and Screening

**Figure 2:**
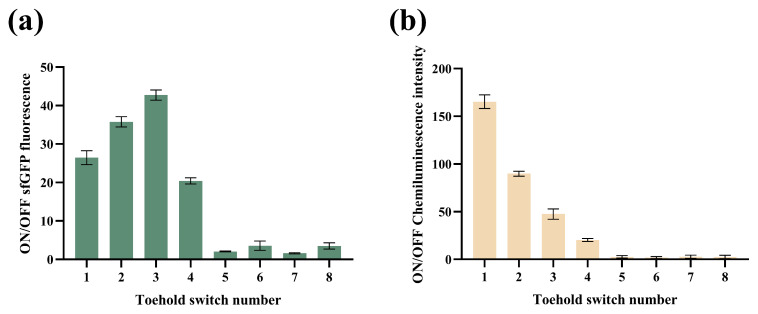
Screen depicting trigger/switch pairs in vivo. (**a**) ON/OFF sfGFP fluorescence ratios of in vivo expression (VPS-G). (**b**) ON/OFF chemiluminescence ratios of in vivo expression (VPS-L).

For trigger RNA design, the hemolysin gene tlh was selected as the target gene because it is widespread in all *V. parahaemolyticus* strains [30,31]. A total of eight unique toehold switches and trigger pair candidates were selected using NUPACK software 3.4.2 [21,22]. Every sensor performance was further evaluated by transforming the toehold switch and trigger pair into *E. coli* BL21 (DE3). This experimental strategy was referenced from Ilkay Cisil Koksaldi [4]. The signal intensity of both the ON and OFF status were measured separately by a toehold switch with or without the presence of a trigger. As shown in Figure 2a, when sfGFP was employed as the reporter gene, S3 performed the highest ON/OFF ratios. Therefore, S3 was selected to construct the *V. parahaemolyticus* toehold switch sensor with sfGFP (hereafter referred to as VPS-G). As shown in Figure 2b, when LuxSit-i was employed as the reporter gene, S1 performed the highest ON/OFF ratios. Therefore, S1 was selected to construct the *V. parahaemolyticus* toehold switch sensor with LuxSit-i (hereafter referred to as VPS-L). For the same switch and trigger pair, VPS-G and VPS-L performed different ON/OFF ratios. This might be due to the secondary structure change brought by different sequence lengths of reporters [32].

### 3.3. Cell-Free System Optimization

**Figure 3:**
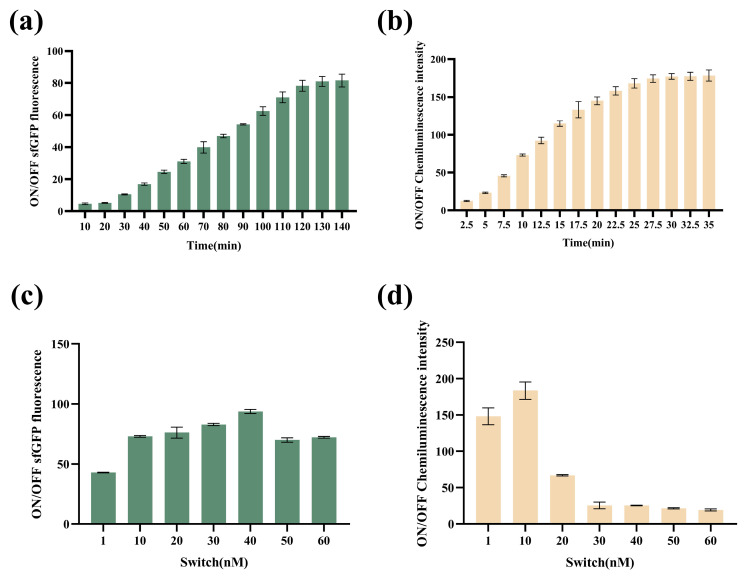
The optimization of the reaction time and concentration of switches in a cell-free system. (**a**) ON/OFF sfGFP fluorescence ratios were monitored as a function of time (VPS-G). (**b**) ON/OFF chemiluminescence ratios were monitored as a function of time (VPS-L). (**c**) ON/OFF sfGFP fluorescence ratios under the different switch concentrations (VPS-G). (**d**) ON/OFF chemiluminescence ratios under the different switch concentrations (VPS-L).

The linear DNA encoding switch sequence was mixed with a related reagent, and then, the switch RNA was generated by transformation. When the target trigger RNA was present, the reporter gene was translated to generate a signal output. The VPS-G fluorescent signal over time was shown in Figure 3a, and the maximum ON/OFF fluorescence ratios remained stable after 2 h. For VPS-L, the chemiluminescence signal reached a plateau after 0.5 h, as shown in Figure 3b. Thus, the VPS-G best signal was obtained at 2 h, and that for VPS-L was at 0.5 h.

To determine optimal switch concentration in biosensors, a series of switch concentrations were tested in VPS-G and VPS-L, respectively. As depicted in Figure 3c, the ON/OFF sfGFP fluorescence reached the highest point with the 40 nM switch. As for VPS-L, the ON/OFF chemiluminescence intensity reached the highest point with the 10 nM switch (Figure 3d). Thus, switch concentrations were selected as 40 nM and 10 nM in VPS-G and VPS-L, respectively.

### 3.4. Sensitivity of the Cell-Free Toehold Switch Sensor with RPA

**Figure 4:**
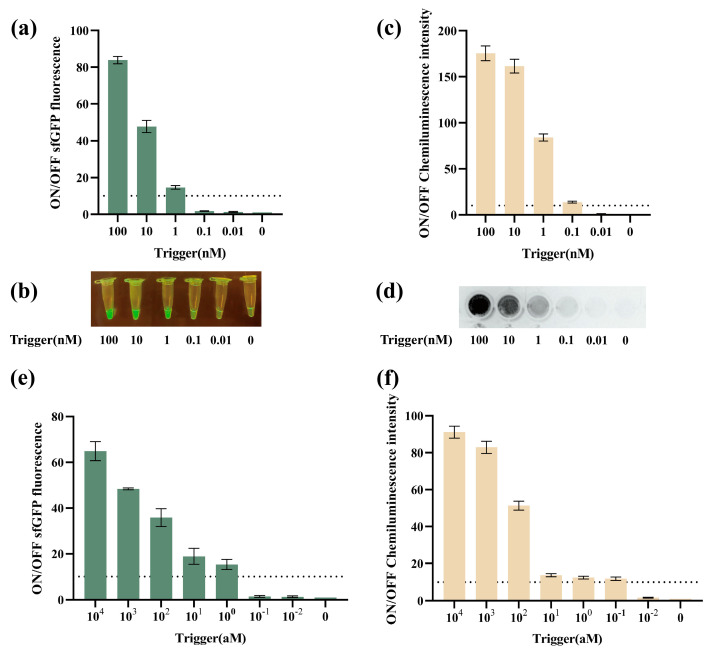
The sensitivity of the cell-free toehold switch sensors combined with RPA. (**a**) ON/OFF sfGFP fluorescence ratios of different concentrations of the trigger (VPS-G). (**b**) The corresponding image for the different triggers of VPS-G. (**c**) ON/OFF chemiluminescence ratios of different concentrations of the trigger (VPS-L). (**d**) The corresponding image for the different triggers of VPS-L. (**e**) ON/OFF sfGFP fluorescence ratios of different concentrations of the trigger with RPA (VPS-G). (**f**) ON/OFF chemiluminescence ratios of different concentrations of the trigger with RPA (VPS-L).

Considering the *V. parahaemolyticus* was a bacteria, RPA was first selected to amplify the target. The principle of RPA was shown in Figure S1. Before amplification, a series of concentrations of *V. parahaemolyticus* target DNA were then prepared to assess the original sensitivity of VPS-G and VPS-L. VPS-G was found to be activated by more than 1 nM trigger DNA (Figure 4a), generating obviously green fluorescence under blue light (Figure 4b). For VPS-L, it could be activated by more than 0.1 nM trigger DNA (Figure 4c), generating an obvious signal that could be observed under a gel imager (Figure 4d). Meanwhile, in order not to rely on expensive instruments to detect the output signal, we attempt to use mobile phone photography to capture the signal, as shown in Figure S2.

Next, the performance of VPS-G and VPS-L with RPA was further evaluated. Trigger DNA was amplified, which was further verified by agarose gel electrophoresis and Qubit 4 fluorometer (Figure S3). A wider range of initial trigger DNA concentrations, ranging from 0 aM to 10^4^ aM, were employed to assess the sensitivity following RPA. The data showed that more than 1 aM trigger DNA could activate VPS-G (Figure 4e). For VPS-L, only 0.1 aM trigger DNA could activate the toehold switch (Figure 4f).

### 3.5. Sensitivity of the Cell-Free Toehold Switch Sensor with NASBA

**Figure 5:**
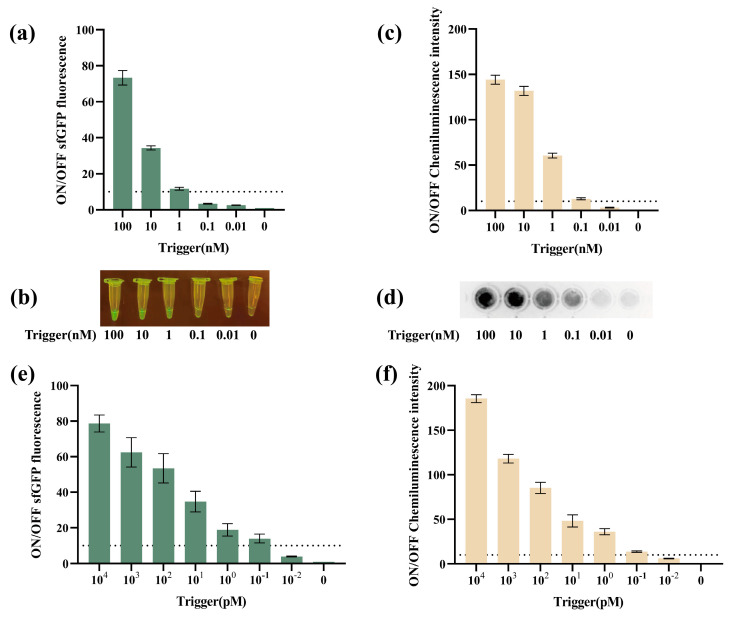
The sensitivity of the cell-free toehold switch sensors combined with NASBA. (**a**) ON/OFF sfGFP fluorescence ratios of different concentrations of the trigger RNA (VPS-G). (**b**) The corresponding image for the different trigger RNA of VPS-G. (**c**) ON/OFF chemiluminescence ratios of different concentrations of the trigger RNA (VPS-L). (**d**) The corresponding image for the different trigger RNA of VPS-L. (**e**) ON/OFF sfGFP fluorescence ratios of different concentrations of the trigger RNA with NASBA (VPS-G). (**f**) ON/OFF chemiluminescence ratios of different concentrations of the trigger RNA with NASBA (VPS-L).

For bacterial detection, molecular biological methods usually faced difficulties in distinguishing between live and dead cells. To overcome this limitation, we try to use NASBA to amplify target RNA in living bacteria. The schematic of NASBA was shown in Figure S4. Here, the NASBA strategy was constructed at the gene level. The different concentrations of RNA came from transcription in vitro. Before NASBA, VPS-G was found to be activated by more than 1 nM trigger RNA (Figure 5a). Furthermore, the production of sfGFP was clearly visible under blue light when the trigger RNA was added at concentrations exceeding 1 nM (Figure 5b). In the case of VPS-L, it was observed that more than 0.1 nM trigger RNA could activate a signal output (Figure 5c). Similarly, the luminescence output was clearly visible under a gel imager (Figure 5d). These activation trigger RNA concentrations were consistent with trigger DNA concentrations.

Next, the effectiveness of VPS-G and VPS-L when combined with NASBA was further assessed. The trigger RNA was amplified, which was further verified by agarose gel electrophoresis and Qubit 4 fluorometer (Figure S5). As depicted in Figure 5e, the VPS-G was activated by more than 0.1 pM original RNA after NASBA. For VPS-L, the activation original trigger RNA concentration was also 0.1 pM after NASBA (Figure 5f).

### 3.6. Cell-Free Toehold Switch Sensor Performance on V. parahaemolyticus

**Figure 6:**
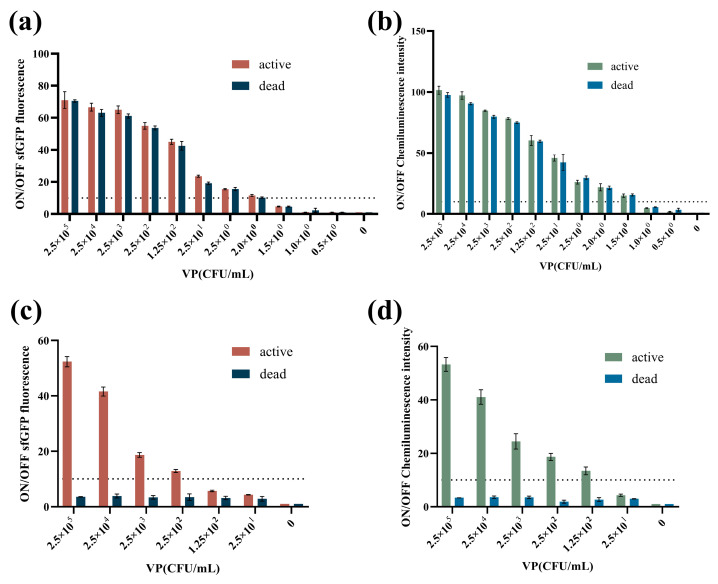
Toehold switch sensor performance on *V. parahaemolyticus*. (**a**) ON/OFF fluorescence ratios of VPS-G for the detection of both dead and live *V. parahaemolyticus* with RPA. (**b**) ON/OFF chemiluminescence ratios of VPS-L for the detection of both dead and live *V. parahaemolyticus* with RPA. (**c**) ON/OFF fluorescence ratios of VPS-G for the detection of both dead and live *V. parahaemolyticus* with NASBA. (**d**) ON/OFF chemiluminescence ratios of VPS-L for the detection of both dead and live *V. parahaemolyticus* with NASBA.

To investigate VPS-G and VPS-L sensitivity of detecting *V. parahaemolyticus*, a series of concentrations of *V. parahaemolyticus* bacterium solution was used as the target for testing. Here, live and dead bacteria were, respectively, tested via RPA or NASBA. First, VPSs detection performance with RPA was evaluated. As shown in Figure 6a, the results indicated that VPS-G was activated by at least 2.0 CFU/mL to produce sfGFP to emit the fluorescence, whether in live or dead *V. parahaemolyticus*. For VPS-L, a detectable signal was generated by more than 1.5 CFU/mL, both in live and dead *V. parahaemolyticus* (Figure 6b).

Then VPSs’ detection performance with NASBA was evaluated. As shown in Figure 6c, only live *V. parahaemolyticus* could activate VPS-G at a concentration of at least 250 CFU/mL, and dead *V. parahaemolyticus* could not. Similarly, more than 125 CFU/mL live *V. parahaemolyticus* could activate VPS-L, whereas the dead ones could not (Figure 6d). The results showed that the cell-free toehold switch-combined NASBA reactions could identify live bacterial existence.

### 3.7. Specificity of Cell-Free Toehold Switch Sensor

**Figure 7:**
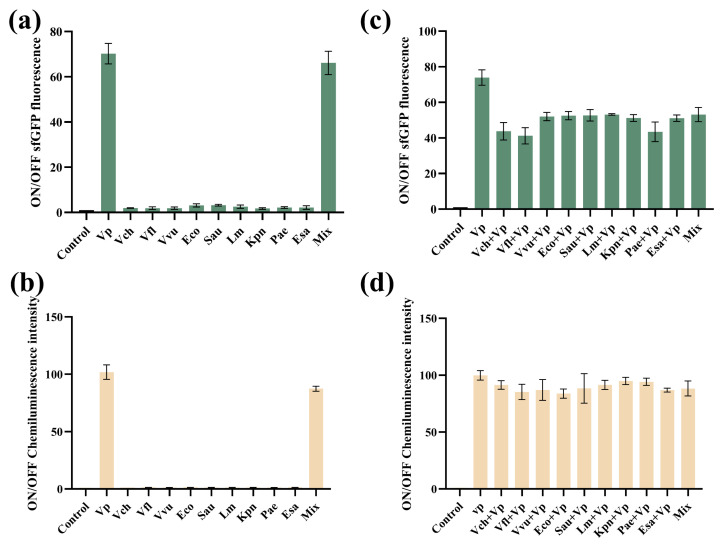
The specificity of the cell-free toehold switch sensor for *V. parahaemolyticus*. (**a**) ON/OFF sfGFP fluorescence ratios of the different strains (VPS-G). (**b**) ON/OFF chemiluminescence ratios of the different strains (VPS-L). (**c**) ON/OFF sfGFP fluorescence ratios of the different mixed strains (VPS-G). (**d**) ON/OFF chemiluminescence ratios of the different mixed strains (VPS-L). Mix represents all test samples/bacteria mixture.

In order to evaluate the specificity of VPS-G and VPS-L, several common pathogenic bacteria were tested, including *vibrio fluvialis*, *vibrio cholerae*, *vibrio vulnificus*, *escherichia coli*, *staphylococcus aureus*, *listeria monocytogenes*, *klebsiella pneumoniae*, *pseudomonas aeruginosa*, and *enterobacter sakazaki*. As shown in Figure 7a,b, only *V. parahaemolyticus* could lead to the positive result, demonstrating the high specificity. Moreover, VPS-G and VPS-L could be correctly triggered by the target, even if *V. parahaemolyticus* was mixed with other pathogenic bacteria (Figure 7c,d).

## 4. Discussion

The present study demonstrates that combining toehold switch sensors and RPA or NASBA in a cell-free system can effectively detect *V. parahaemolyticus* with high sensitivity and specificity.

The optimal switch concentrations for VPS-G and VPS-L were 40 nM and 10 nM, respectively. These values are in line with prior research in related fields, such as those for SARS-CoV-2 [4], Coronaviruses [3], and norovirus detections [6,33]. The variation in concentrations between VPS-G and VPS-L may stem from their different switch–trigger pairs. For VPS-G, the maximum ON/OFF sfGFP fluorescence ratio was 80 fold, comparable to the 60 fold ratios in Zika virus [2] and IP10 mRNA detections [9]. Our study shows an advantage in this ratio compared to previous works on norovirus [6], SARS-CoV-2 [4], and Coronavirus detections [3]. Regarding VPS-L, at an additional concentration of 10 nM, the maximum ON/OFF chemiluminescence intensity reached 180 fold. Clearly, LuxSit-i significantly boosts the trigger response value compared to sfGFP, which is a strength over previous studies. Overall, regardless of the reporter gene used, these results confirm the successful construction of the toehold switch for *V. parahaemolyticus* detection.

Sensitivity is an important indicator for evaluating testing techniques. The detection limit of VPS-G and VPS-L before amplification is 1 nM or 0.1 nM. Interestingly, in the case of detecting coronaviruses [3], the activation of the toehold switch before amplification requires a concentration of 250 nM for the trigger RNA. On the other hand, when it comes to Zika virus [2] detection, a concentration as low as 30 nM for the trigger RNA is sufficient to activate the toehold switch before NASBA. The variance in minimum detection concentrations could be attributed, at least in part, to the different sequence-dependent free energy present in each case. After amplification, the detection level can reach the aM level. In previous reports, after amplified, the detection limits were 22 fM for Norovirus [6], 200 aM for Norovirus [6], 91 aM and 5.2 fM for integrated respiratory virus subgroups B and A [5], and 3 fM for Zika virus [2]. The research findings are consistent with previous studies and demonstrate even lower detection limits.

In previous studies, the best incubation time in toehold switch sensors was typically around 2 h when using GFP or LacZ as the reporter gene [2,5,17]. In our study, we found that for the VPS-G sensor (using sfGFP as the reporter), the optimal incubation time was also 2 h. However, when it comes to the VPS-L sensor (using LuxSit-i as the reporter), the optimal incubation time was only 0.5 h. This significant difference in the optimal incubation time between VPS-G and VPS-L can be mainly attributed to the characteristics of the two reporters. The luciferase LuxSit-i, designed by Andy Hsien Wei Yeh [19], is a relatively small protein with a molecular weight of 13.9 kDa and a gene sequence length of 354 bp. In contrast, sfGFP has a gene length of 720 bp and a molecular weight of 27 kDa. Since the LuxSit-i gene is approximately half the length and molecular weight of sfGFP, it takes less time to be synthesized. The shorter synthesis time of LuxSit-i compared to sfGFP directly leads to a shorter optimal incubation time in the VPS-L sensor. This application of LuxSit-i in the VPS-L sensor significantly reduces the incubation time, which is a great advantage, as it can accelerate the entire detection process.

DNA can persist in the environment, resulting in extracellular DNA and DNA from dead cells that is indistinguishable from DNA representing living cells [34,35,36]. Compared with DNA, RNA is only found in living cells [15,16]. NASBA is an RNA template for amplification under constant temperature conditions. Therefore, the question is whether NASBA can be used in a cell-free detection system to amplify RNA and distinguish between dead and live bacteria. To address this, a genomic-level detection system combining toehold switch and NASBA was established. The results showed that after NASBA, only live *V. parahaemolyticus* could activate VPS-G (at a concentration of 250 CFU/mL) and VPS-L (at 125 CFU/mL), while dead bacteria could not. However, after RPA, both live and dead *V. parahaemolyticus* could activate VPS-G (at least 2.0 CFU/mL) and VPS-L (at least 1.5 CFU/mL). The results indicate that we can judge whether there are viable bacteria according to this result.

*V. parahaemolyticus* is a common foodborne pathogenic microorganism, causing food poisoning outbreaks and raising significant public health concerns. Recently, the toehold switch has been incorporated into nucleic acid detection systems, and there have been several reviews on its application in detection. Our study introduced two key innovations compared to previous research. First, in terms of toehold switch applications, it was originally used for detecting RNA viruses, but in our study, it has been applied to detect bacteria. Moreover, by combining the toehold switch with RPA and NASBA, respectively, we achieved the differentiation between live and dead *V. parahaemolyticus*. Second, we incorporated an artificially designed highly specific LuxSit-i as the reporter gene. This not only ensured a lower detection limit but also significantly reduced the detection time. Based on previous studies, we developed a new toehold switch assay for *V. parahaemolyticus*. This assay is fast, sensitive, simple, and accurate. Consequently, the toehold switch-based detection platform is suitable for rapidly detecting foodborne pathogens, which is of great significance for food safety management, the prevention of foodborne diseases, and public health.

## Data Availability

Data will be made available on request.

## Supplementary Materials

The following supporting information can be downloaded at https://www.mdpi.com/article/10.3390/bios14120637/s1, Figure S1: The schematic of RPA reaction; Figure S2: Luminescence images acquired by a Huawei smartphone camera. Tubes from left to right: DTZ; None DTZ; Figure S3: Validation of RPA efficiency by agarose gel electrophoresis and concentration determination; Figure S4: The schematic of NASBA reaction; Figure S5: Validation of NASBA efficiency by agarose gel electrophoresis and concentration determination; Table S1: Sequences of 8 pairs of toehold switch sensors and corresponding target trigger; Table S2: The primer sequence for the experiment; Table S3: The sequence of output signals for the experiment.
