# Supplementary material for: A Novel Approach Using LuxSit-i Enhanced Toehold Switches for the Rapid Detection of Vibrio parahaemolyticus
Source: Biosensors (Basel). 2024 Dec 21;14(12):637. doi: 10.3390/bios14120637 (PMC11674225; doi:10.3390/bios14120637)

biosensors

MDPI

Article

A novel approach using LuxSit-i enhanced toehold switches for the rapid detection of Vibrio parahaemolyticus.

Xiaodan Kang [1,2,+], Chen Zhao [1,+], Shuting Chen [1,2], Shuran Yang [1], Xi Zhang [1], Bin Xue [1], Chenyu Li [1], Shang Wang [1], Xiaobo Yang [1], Chao Li [1], Zhigang Qiu [1], Jingfeng Wang [1,*] and Zhiqiang Shen [1,*]

Supplementary materials

Table S1 Sequences of 8 pairs toehold switch sensors and corresponding target trigger

	Target sequence	Sensor sequence
1	ACAATCACACTATTAACTGCAT TACTCCCGCTTGCT	GGGAGCAAGCGGGAGTAATGCAGTTAA***TAGTGTGATTGT***GGACTTTAG AACAGAGGAGATAAAGATG***ACAATCACACT***AAACCTGGCGGCAGCGC AAAAG
2	AAACAATCACACTATTAACTGC ATTACTCCCGCTTG	GGGCAAGCGGGAGTAATGCAGTTAATA***GTGTGATTGTTT***GGACTTTAG AACAGAGGAGATAAAGATG***AAACAATCACAC***AACCTGGCGGCAGCGC AAAAG
3	ATCACACTATTAACTGCATTAC TCCCGCTTGCTTCT	GGGAGAAGCAAGCGGGAGTAATGCAGT***TAATAGTGTGAT***GGACTTTAG AACAGAGGAGATAAAGATG***ATCACACTATTA***AACCTGGCGGCAGCGCA AAAG
4	CAATCACACTATTAACTGCATT ACTCCCGCTTGCTT	GGGAAGCAAGCGGGAGTAATGCAGTTA***ATAGTGTGATTG***GGACTTTAG AACAGAGGAGATAAAGATG***CAATCACACTAG***AACCTGGCGGCAGCGC AAAAG
5	CACACTATTAACTGCATTACTC CCGCTTGCTTCTGC	GGGGCAGAAGCAAGCGGGAGTAATGCA***GTTAATAGTGTG***GGACTTTAG AACAGAGGAGATAAAGATG***CAATCACACTAG***AACCTGGCGGCAGCGC AAAAG
6	GAATGCAGGAAGCCATATAAA GAATTGAGATTAGAA	GGGTTCTAATCTCAATTCTTTATATGG***CTTCCTGCATTC***GGACTTTAGAA CAGAGGAGATAAAGATG***CACACTATTAAC***AACCTGGCGGCAGCGCAA AAG
7	CGCCGCTGACAATCGCTTCTCA TACAACCACACGAT	GGGATCGTGTGGTTGTATGAGAAGCGA***TTGTCAGCGGCG***GGACTTTAG AACAGAGGAGATAAAGATG***CGCCGCTGACAA***AACCTGGCGGCAGCGC AAAAG
8	CGAAGAACTACAAACCAGCAA ACACCTTGTTTACGC	GGGCGTAAACAAGGTGTTTGCTGGTT***TGTAGTTCTTCG***GGACTTTAGAA CAGAGGAGATAAAGATG***CGAAGAACTACA***AACCTGGCGGCAGCGCAA AAG

Notes: Sequence regions in complementarity between target and senser are underlined, and base pair forming regions in sensor RNA to form stem are shown in italic.

Table S2 The primers sequence for the experiment

Purpose	sequence
Switch-F	ACGCCAAGCTTTAATACGAC
Switch-R	TTCAGCAAAAAACCCCTCAA
Trigger-F	GGTGCGATACCAACAGC
Trigger-R	TAATACGACTCACTATAGGGGATG
NASBA-F	GTGCGATACCAACAGCGAACATAGG
NASBA-R	AATTCTAATACGACTCACTATAGGGGATGA
RPA-F	CGAGTAGCTGGTGCGATACCAACAGCGAAC
RPA-R	GCTAATACGACTCACTATAGGGGATGAAAA

Table S3 The sequence of output signals for the experiment

	DNA sequence
sfGFP	ATGAGCAAAGGAGAAGAACTTTTCACTGGAGTTGTCCCAATTCTTGTTGAATTA GATGGTGATGTTAATGGGCACAAATTTTCTGTCCGTGGAGAGGGTGAAGGTGAT GCTACAAACGGAAAACTCACCCTTAAATTTATTTGCACTACTGGAAAACTACCT GTTCCGTGGCCAACACTTGTCACTACTCTGACCTATGGTGTTCAATGCTTTTCCC GTTATCCGGATCACATGAAACGGCATGACTTTTTCAAGAGTGCCATGCCCGAAG GTTATGTACAGGAACGCACTATATCTTTCAAAGATGACGGGACCTACAAGACGC GTGCTGAAGTCAAGTTTGAAGGTGATACCCTTGTTAATCGTATCGAGTTAAAGG GTATTGATTTTAAAGAAGATGGAAACATTCTTGGACACAAACTCGAGTACAACT TTAACTCACACAATGTATACATCACGGCAGACAAACAAAAGAATGGAATCAAA GCTAACTTCAAAATTCGCCACAACGTTGAAGATGGTTCCGTTCAACTAGCAGAC CATTATCAACAAAATACTCCAATTGGCGATGGCCCTGTCCTTTTACCAGACAAC CATTACCTGTCGACACAATCTGTCCTTTCGAAAGATCCCAACGAAAAGCGTGAC CACATGGTCCTTCTTGAGTTTGTAACTGCTGCTGGGATTACACATGGCATGGATG AGCTCTACAAA
LuxSit-i	ATGGGTAGCCATCACCACCATCACCATGGTAGCGGTAGCGAGAACTTGTACTTC CAAGGAATGAGCGAAGAACAGATTCGTCAGTTTCTGCGTCGTTTTTATGAAGCG CTGGATAGCGGCGATGCGGATACCGCTGCGAGCCTGTTTCATCCGGGCGTGACA ATTCATCTGTGGGATGGCGTTACCTTTACCAGCCGTGAAGAATTTCGTGAATGGT TTGAACGTCTGTTTAGCACCAGTAAAGATGCGCAGCGTGAAATTAAGAGCCTGG AAGTACGTGGCGATACCGTGGAAGTGCATGTGCAGTTGCACGCGACCCATAAT GGCCAGAAACATACCGTAGATTTGACCCATCATTGGCATTTTCGTGGCAATCGT GTGACCGAAGTTCGTGTGCATATCAATCCGACCGG

Figure S1 The schematic of RPA reaction.

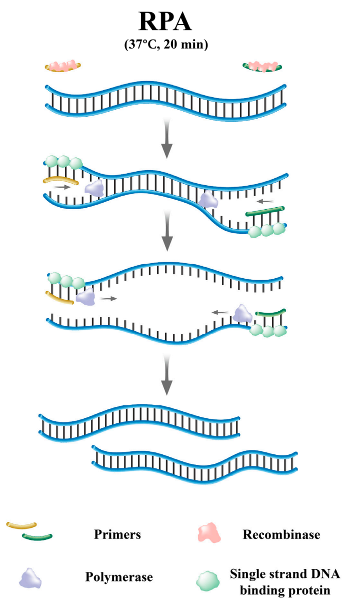

Figure S2 Luminescence images acquired by a Huawei smartphone camera. Tubes from left to right: DTZ; None DTZ.

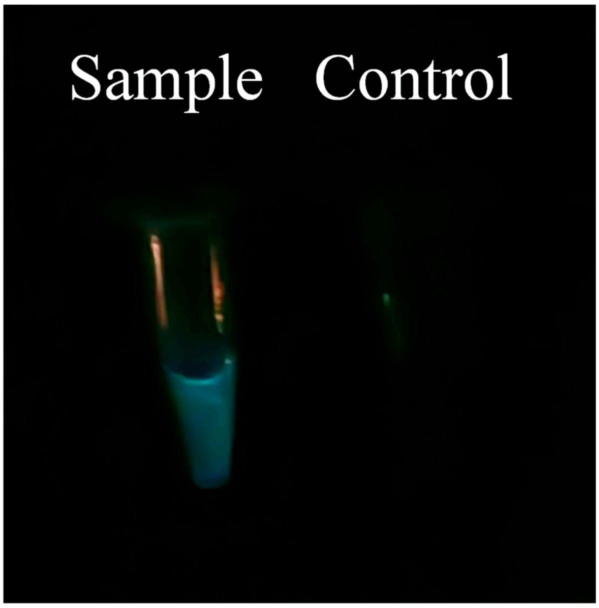

Figure S3 Validation of RPA amplification efficiency by agarose gel electrophoresis and concentration determination. (a) Marker: In gel electrophoresis, a Marker (molecular marker) is a standard reference with known molecular weights. Control: the control samples consist of the target DNA except all the amplification reaction components. RPA: the control samples consist of the target DNA and the amplification reaction components. (b) The concentration measurement of the sample after the reaction of control and RPA in Fig S2(a).

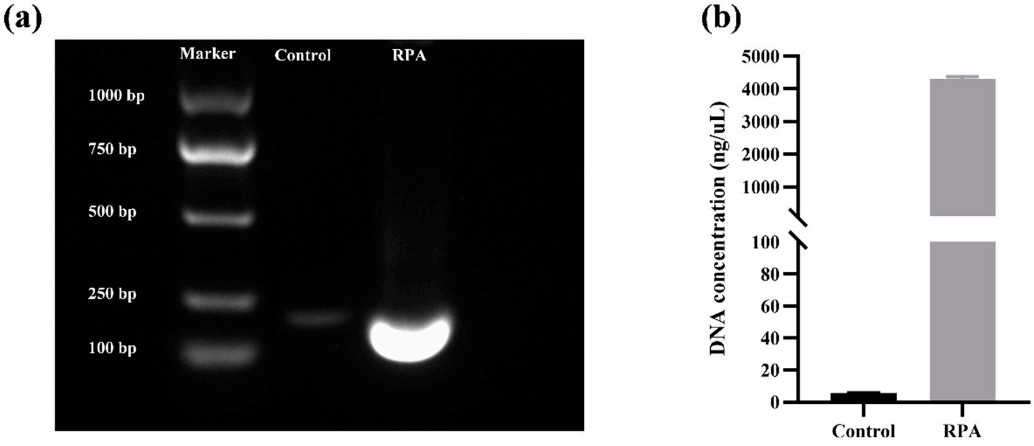

Figure S4 The schematic of NASBA reaction.

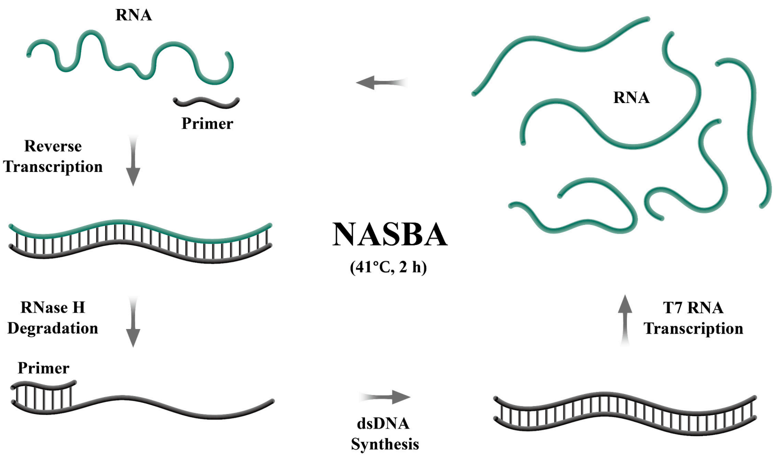

Figure S5 Validation of NASBA amplification efficiency by agarose gel electrophoresis and concentration determination. (a) Marker: In gel electrophoresis, a Marker (molecular marker) is a standard reference with known molecular weights. Control: the control samples consist of the target RNA except all the amplification reaction components. NASBA: the control samples consist of the target RNA and the amplification reaction components. (b) The concentration measurement of the sample after the reaction of control and RPA in Fig S3(a).

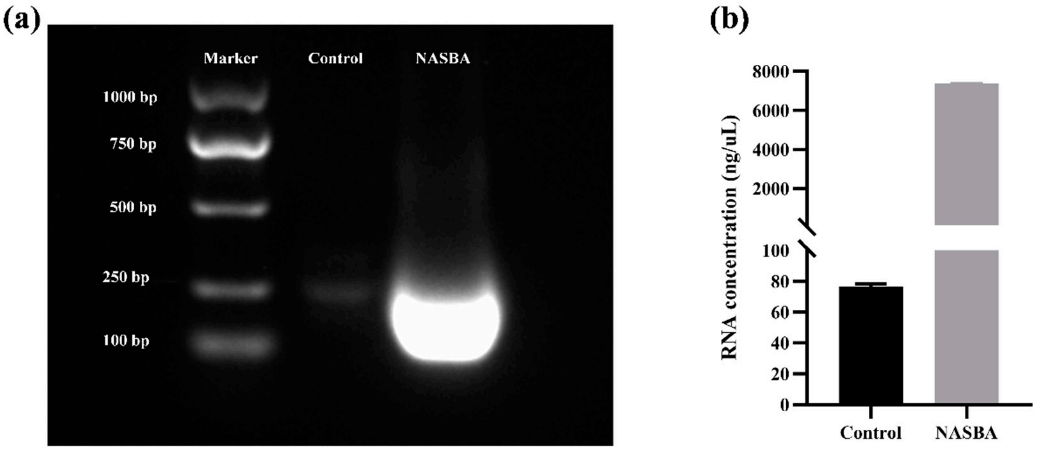

Supplement: Supplementary file 1 [file biosensors-14-00637-s001.zip › biosensors-3302106-supplementary.pdf]
